# Association of Pro12Ala polymorphism in peroxisome proliferator activated receptor gamma with proliferative diabetic retinopathy

**Published:** 2013-03-21

**Authors:** Khadija Tariq, Saira Bano Malik, Syeda Hafiza Benish Ali, Sundas Ejaz Maqsood, Aisha Azam, Irfan Muslim, Muhammad Shakil Khan, Maleeha Azam, Nadia Khalida Waheed, Raheel Qamar

**Affiliations:** 1 Department of Biosciences, Faculty of Science, COMSATS Institute of Information Technology, Islamabad, Pakistan; 2 Institute of Pure and Applied Biology, Bahauddin Zakariya University, Multan, Pakistan; 3 Department of Ophthalmology, Shifa International Hospital, Islamabad, Pakistan; 4 Institute of Ophthalmology, Mayo Hospital, Lahore, Pakistan; 5 Al-Nafees Medical College & Hospital, Isra University, Islamabad, Pakistan; 6 Tufts University Medical School, Boston, MA

## Abstract

**Purpose:**

The association of non-synonymous substitution polymorphism rs1801282 (c.34C>G, p.Pro12Ala) in exon 4 of the peroxisome proliferator activated receptor gamma gene with diabetic retinopathy (DR) has been reported inconsistently. Therefore, the purpose of the present study was to understand the population-specific role of the Pro12Ala polymorphism in DR susceptibility in Pakistani subjects.

**Methods:**

A total of 180 subjects with DR, 193 subjects with type 2 diabetes mellitus (T2DM) with no diabetic retinopathy, and 200 healthy normoglycemic non-retinopathic Pakistani individuals were genotyped for the rs1801282 (c.34C>G) polymorphism using polymerase chain reaction-restriction fragment length polymorphism.

**Results:**

We found the individuals with T2DM carrying 12Ala were at a reduced risk of developing DR (odds ratio [OR]=0.53; 95% confidence interval [CI]=0.33–0.87). Upon stratified analysis regarding disease severity, we observed this protective effect was confined to proliferative DR (OR=0.4; 95% CI=0.2–0.8) with non-significant effects on the susceptibility of non-proliferative DR (OR=0.67; 95% CI=0.37–1.19).

**Conclusions:**

We report a protective role of the 12Ala polymorphism against proliferative DR in individuals with T2DM in Pakistan.

## Introduction

Diabetic retinopathy (DR), a microangiopathy resulting from chronic hyperglycemia [1], affects almost 100% of type 1 diabetes mellitus (T1DM) patients and around 77% of T2DM-affected individuals who have had the disease for at least 20 years [2]. The earlier stage of the disease is termed non-proliferative diabetic retinopathy (NPDR) and is characterized by increased vascular permeability and vascular closure. In the later stage, the disease progresses to proliferative diabetic retinopathy (PDR), a severe condition characterized by proliferation of new blood vessels in the retina and severe visual loss leading to diabetes-induced blindness [3]. The retina is one of the prime insulin targets where insulin mediates a prosurvival pathway in the retinal neurons. In addition, the normal retina also expresses a highly active basal insulin receptor/Akt signaling pathway that is stable in feeding and fasting [4]. Thus, disturbances in these physiologic processes due to insulin resistance have previously been implicated in DR pathology specifically in individuals with T2DM [5]. Insulin resistance has been significantly influenced by genetic factors; therefore, it is biologically plausible to study the association of DR with polymorphisms in genes affecting insulin resistance. One such gene is peroxisome proliferator activated receptor gamma (*PPARγ*), encoding the PPARγ protein, a member of the PPAR subfamily belonging to the nuclear hormone receptor super-family with two other members PPARα and PPARβ/δ. Only the role of PPARγ in DR pathogenesis has been elucidated mainly because of the protein’s role in vascular permeability, inflammation, angiogenesis, neovascularization, and insulin resistance, all of which contribute to the onset and severity of DR [5-8].

The members of the nuclear hormone receptor sub-family have three domains: a ligand-independent activation domain, a DNA-binding domain, and a ligand-activated domain [9]. The two functional isoforms of PPARγ, namely, γ1 and γ2, have similar DNA-binding and ligand-activated domains but differ by an additional 30 amino acids at the N-terminal of γ2 [10]. Unlike γ1, which is expressed in various tissues, γ2 expression is restricted to adipose tissue [11] where expression is stimulated by insulin action [12]. A non-synonymous genetic polymorphism Pro12Ala (rs1801282) in the ligand-independent domain of PPARγ2 is the most extensively studied polymorphism because it renders the receptor more sensitive to insulin action [13]. Several studies have shown decreased insulin resistance and increased insulin sensitivity in 12Ala carriers [14-17]. Through an unknown mechanism, this polymorphism has been linked to T2DM [18] and its complications including diabetic nephropathy [19,20], but some studies have reported contradictory results [13,21]. Another study reported the Pro12Ala polymorphism was protective against diabetes in Caucasians but not in South Asians [22]. Therefore, the role of this polymorphism in DR etiology is still obscure.

To date, no genetic association studies have been conducted on individuals with DR in Pakistan. The aim of the present study was to determine the association of Pro12Ala in *PPARγ2* with the onset and severity of DR in individuals with T2DM.

## Methods

### Sample collection and DNA isolation

The present study was approved by the department ethics committee of the collaborating institutes and conformed to the tenets of the Declaration of Helsinki. Subjects who had T2DM for at least 10 years were recruited from collaborating hospitals. The participants underwent an ophthalmological examination, including visual acuity, slit lamp examination, and funduscopy for the absence or presence of retinopathy. Inclusion criteria, based on the American Diabetes Association criteria on diagnosis of T2DM, were age 18–75 years, fasting plasma glucose level ≥126 mg/dl, random plasma glucose concentration ≥200 mg/dl, serum creatinine concentration ≤2.0 mg/dl, and glycosylated hemoglobin below 11% [10]. Those who showed clinical symptoms of retinopathy were classified as the DR group, and the rest were designated as DNR. The DR group was further classified into NPDR based on the presence of retinal microaneurysms, dot and blot or flame-shaped hemorrhages, hard exudates, cotton wool spots, and intraretinal microvascular abnormalities, and into PDR based on retinal neovascularization with or without preretinal hemorrhage, or fibrous proliferation (representing regressed neovascularization). In addition to the individuals with T2DM, healthy normoglycemic non-retinopathic (NGNR/control) individuals were also recruited for the study. The inclusion criteria for the healthy controls were individuals who were negative for diabetes, hypertension, myocardial infarction, cancer, and retinopathy independent of diabetes.

After informed written consent was obtained, venous blood was collected from 180 individuals with DR, 193 individuals with DNR, and 200 controls (NGNR). Genomic DNA was extracted by a standard phenol/chloroform method of DNA isolation [23]. Briefly, the protocol consisted of cell lysis and protein digestion followed by phenol-chloroform extraction of the peptides, and precipitation of DNA with ethanol. The precipitated DNA was resuspended in Tris-EDTA buffer and stored at 4 °C before use.

### Pro12Ala genotyping

Genotyping was done with the polymerase chain reaction-restriction fragment length polymorphism (PCR-RFLP) method using Yen et al.’s primers [24]. Briefly, the 267 bp amplified fragment was subjected to restriction digestion by *Bst*UI, which resulted in digestion of the PCR product into two fragments of 224 and 43 bp length when the polymorphic site had the G allele (Ala), while the PCR amplicon remained uncut in the presence of the C allele (Pro).

Genotyping was further validated by replicating 10% of the samples at random with *Bst*UI digestion and another 10% of the samples with HpaII digestion. The latter also has a restriction site at this sequence, but the cleavage pattern is opposite to that of *Bst*UI, i.e., it cuts the DNA fragment in the presence of the C allele while the G allele remains uncut. The results of the replicated samples were 100% concordant to the original results.

### Statistical analysis

The Student *t* test was applied using EpiCalc software to compare the average age of the three study groups. Deviation from Hardy–Weinberg equilibrium of genotype frequencies in the control individuals was tested with the goodness-of-fit χ^2^ test, while the χ^2^ test of independence was computed using StatCalc software to compare the genotype and allele frequencies between study groups. Logistic regression analysis was performed to determine the association of the Pro12Ala variant with disease onset and severity by using EpiCalc software.

## Results

Ethnic background and average age were matched between the study groups consisting of 180 individuals with DR, 193 individuals with DNR, and 200 controls (NGNR; p>0.05, data not shown). Out of the 180 subjects with DR, half (n=90) had NPDR, and the other half were diagnosed with PDR. The genotype frequency of the control (NGNR) group was consistent with Hardy–Weinberg equilibrium.

In the present study, the frequency of the 12Ala homozygotes was highest in the DNR group (overall: n=8 [4%], male: n=6 [6.4%], female: n=2 [2%]) followed by controls (n=2 [1%]; both of whom were male), while no 12Ala homozygote was found in the DR group (Table 1). Thus, logistic regression analysis was performed assuming a dominant model for 12Ala for genotype association, and allele frequencies were compared. Analysis of the DR and control subjects showed no significant association (p>0.05), although genotype frequency distribution was significantly different (p<0.05) among women. Similarly, there was no genotype or allele association in a comparison between the DNR group and the controls. A significant protective effect (p<0.05) of 12Ala was observed in a DR versus DNR comparison after adjusting for age and gender in an overall analysis, which diminished in the gender-based data analysis (p>0.05; Table 1). To determine the association of the variant with T2DM irrespective of the presence or absence of microvascular complication, a comparison of subjects with T2DM (DR+DNR) was performed against the control group, which revealed a non-significant association (Table 2).

**Table 1:** Genotype and allele frequency distribution of Pro12Ala polymorphism of PPARγ among DR, DNR, and control groups.

Overall	**Controls**	**DR**	**DNR**	**DR versus Controls**		**DR versus DNR**		**DNR versus Controls**	
	n=200	n=180	n=193	χ^2^ (p)	OR(95%CI)	χ^2^ (p)	OR(95%CI)	χ^2^ (p)	OR(95%CI)
Pro/Pro	155(77.5%)	149(82.8%)	143(74.1%)	3.02	DM=0.72(0.42–1.23)	**9.34**	DM=0.6(0.35–1.01)	3.97	DM=1.2(0.76–1.91)
Pro/Ala	43(21.5%)	31(17.2%)	42(21.8%)	(0.22)		**(0.01)**		(0.14)	
Ala/Ala	2(1%)	0	8(4.2%)						
Pro	353(88%)	329(91.4%)	328(85%)	2.03	0.71(0.43–1.17)	**7.3**	**0.53(0.33–0.87)**	1.82	1.33(0.86–2.05)
Ala	47(12%)	31(8.6%)	58(15%)	(0.15)		**(0.01)**		(0.18)	
**Males**	n=100	n=87	n=94						
Pro/Pro	81(81%)	69(79.3%)	68(72.3%)	2.09	DM=1.11(0.51–2.42)	**5.85**	DM=0.68(0.32–1.43)	3.19	DM=1.63(0.79–3.38)
Pro/Ala	17(17%)	18(20.7%)	20(21.3%)	(0.35)		**(0.05)**		(0.2)	
Ala/Ala	2(2%)	0	6(6.4%)						
Pro	179(89.5%)	156(89.7%)	156(83%)	0.00	0.98(0.48–2.01)	3.38	0.56(0.29–1.09)	3.49	1.75(0.93–3.29)
Ala	21(10.5%)	18(10.3%)	32(17%)	(0.96)		(0.07)		(0.06)	
**Females**	n=100	n=93	n=99						
Pro/Pro	74(74%)	80(86%)	75(75.8%)	**4.32**	DM=0.48(0.23–1.05)	4.29	DM=0.51(0.23–1.13)	2.34	DM=0.91(0.46–1.8)
Pro/Ala	26(26%)	13(14%)	22(22.2%)	**(0.04)**		(0.12)		(0.31)	
Ala/Ala	0	0	2(2%)						
Pro	174(87%)	173(93%)	172(87%)	**3.83**	0.52(0.24–1.06)	**3.97**	0.5(0.23–1.05)	0.00	1.01(0.54–1.88)
Ala	26(13%)	13(7%)	26(13%)	**(0.05)**		**(0.05)**		(0.97)	

Pro: C allele; Ala: G allele; χ^2^: Chi-square test of independence; OR(95%CI): Odds Ratio (95% Confidence Interval); DM: Dominant Model (Pro/Ala+Ala/Ala versus Pro/Pro); Statistically significant values (p≤0.05) are written in bold.

**Table 2:** Association of Pro12Ala polymorphism of PPARγ with the risk of T2DM.

	**T2DM**	**T2DM versus Controls**	
	n=373	χ^2^ (p)	OR(95%CI)
Pro/Pro	292(78.3%)	1.23	DM=0.96(0.62–1.48)
Pro/Ala	73(19.6%)	(0.54)	
Ala/Ala	8(2.1%)		
Pro	657(88%)	0.01	1.02(0.69–1.51)
Ala	89(12%)	(0.93)	
**Males**	n=181		
Pro/Pro	137(75.7%)	2.48	DM=1.37(0.72–2.62)
Pro/Ala	38(21%)	(0.29)	
Ala/Ala	6(3.3%)		
Pro	312(86%)	1.28	1.37(0.77–2.44)
Ala	50(14%)	(0.26)	
**Females**	n=192		
Pro/Pro	155(80.7%)	3.32	DM=0.68(0.37–1.25)
Pro/Ala	35(18.2%)	(0.19)	
Ala/Ala	2(1.04%)		
Pro	345(90%)	1.08	0.76(0.43–1.33)
Ala	39(10%)	(0.3)	

Pro: C allele; Ala: G allele; χ^2^: Chi-square test of independence; OR(95%CI): Odds Ratio (95% Confidence Interval); DM: Dominant Model (Pro/Ala+Ala/Ala versus Pro/Pro)

In further analysis, the DR group was divided into two subsets, NPDR and PDR, and each was independently analyzed against the DNR group and the control group (Table 3). The 12Ala allele was not associated with NPDR in both comparisons. The analysis of the PDR subgroup also showed no association with 12Ala compared with the control NGNR group, while a significant protective effect (p<0.05) of the variant was seen compared to the DNR group. A case-only comparison between the PDR and NPDR groups was also performed that showed a non-significant genotype difference between the two sub-groups (Table 3).

**Table 3:** Analysis of association of Pro12Ala polymorphism of PPARγ with NPDR and PDR.

	**NPDR**	**NPDR versus Controls**		**NPDR versus DNR**	
	n=90	χ^2^ (p)	OR(95%CI)	χ^2^ (p)	OR(95%CI)
Pro/Pro	71(79%)	0.92	DM=0.92(0.5–1.69)	3.93	DM=0.77(0.40–1.45)
Pro/Ala	19(21%)	(0.63)		(0.14)	
Ala/Ala	0				
Pro	161(89%)	0.18	0.89(0.48–1.61)	2.09	0.67(0.37–1.19)
Ala	19(11%)	(0.68)		(0.15)	
					
	**PDR**	**PDR versus Controls**		**PDR versus DNR**	
	n=90				
Pro/Pro	78(86.7%)	3.73	DM=0.53(0.23–1.1)	**7.26**	**DM=0.44(0.21–0.91)**
Pro/Ala	12(13.3%)	(0.15)		**(0.03)**	
Ala/Ala	0				
Pro	168(93%)	3.51	0.54(0.26–1.08)	**7.91**	**0.4(0.2–0.8)**
Ala	12(7%)	(0.06)		**(0.005)**	
**PDR versus NPDR**					
Pro/Pro	-	1.91	DM=0.57(0.24–1.4)		
Pro/Ala	-	(0.17)			
Ala/Ala	-				
Pro	-	1.73	0.61(0.27–1.36)		
Ala	-	(0.19)			

Pro: C allele; Ala: G allele; χ^2^: Chi-square test of independence; OR(95%CI): Odds Ratio (95% Confidence Interval); DM: Dominant Model (Pro/Ala+Ala/Ala versus Pro/Pro); Statistically significant values (p≤0.05) are written in bold.

## Discussion

PPARγ, a nuclear receptor, has a major role in transcriptional regulation of genes involved in various physiologic processes of clinical significance such as adipogenesis, inflammation, and angiogenesis. The non-synonymous single nucleotide polymorphism (SNP) rs1801282 (c.34C>G, p.Pro12Ala) in exon 4 of the PPARγ2 isoform has been associated with the etiology of T2DM and its complications. However, the association with DR has been inconsistent; therefore, we studied this SNP in Pakistani subjects with T2DM. The frequency of the 12Ala allele in the current study was low (12%) in accordance with the frequency in other populations, e.g., highest (about 12%) in Caucasians followed by 10% in Mexican Americans, 8% in Samoans, 3% in African Americans, 2% in Nauruans, and 1% in Chinese (the lowest) [24].

The 12Ala isoform of PPARγ had been found to have a protective role against diabetic nephropathy, another common T2DM complication [25]. In the present analysis, we also found a protective role of the 12Ala variant in the onset of PDR among individuals with T2DM. This finding is consistent with Malecki et al. [26] and contradicts others who found no association of this variant with DR [13,21,27]. In a meta-analysis, the Pro12 isoform conferred a risk for T2DM by 1.25-fold compared to 12Ala [18]. However, in the present study, we did not find a higher prevalence of either isoform in the patients with T2DM compared to the control NGNR group. The 12Ala variant has also been shown to reduce the risk of atherosclerosis in subjects susceptible to diabetes [28] and against myocardial infarction in non-diabetic individuals [29]. Although the existing evidence points to a possible protective role of the 12Ala variant in the development of proliferative diabetic retinopathy, the underlying mechanism of this effect is still unknown. One possible explanation is the relation of this isoform with a reduction in insulin resistance [16], a known predisposing factor for diabetic complications including DR [5]. In a clinical study, Parvanova et al. [30] reported the level of insulin sensitivity was an independent risk factor for PDR and not NPDR, which also corroborate our results of a significant risk reduction, in 12Ala-carrying subjects with T2DM, confined to PDR only, and no association with NPDR.

However, the protective role of PPARγ manifested by its reduced transactivation activity due to the 12Ala variation [14,31] contradicts the known role of PPARγ in disease pathology. For instance, Muranaka et al. [7] showed that upon ligand activation, PPARγ reduces leukostasis and leakage in retinal vasculature. In a streptozotocin-induced diabetic mouse model, the authors observed 2.1 times raised levels of retinal leukostasis and 1.9 times greater retinal leakage in PPARγ^+/−^ mice compared to wild-type, thus demonstrating possible evidence of some intrinsic role being played by PPARγ in DR. The researchers also observed a 0.6-fold reduction each in retinal leukostasis and retinal leakage upon treatment of diabetic mice with PPARγ ligand rosiglitazone. To define the underlying mechanism, the researchers investigated the effect on inflammatory markers and found a reduction in raised intercellular adhesion molecule 1 levels in rosiglitazone-treated mice. This finding highlights the anti-inflammatory actions of PPARγ, which could perhaps provide protection against DR. However, streptozotocin-induced diabetes is a T1DM model [32], while insulin resistance characterizes T2DM. Thus, PPARγ might have different roles in T1DM and T2DM. In addition, since 12Ala is found in the ligand-independent domain, the effects are independent of the presence or absence of PPARγ ligands [31,33], e.g., rosiglitazone. Therefore, improvement in insulin sensitivity might be the defining factor, implicating the protective effect of the 12Ala variant against disease proliferation in individuals with T2DM who have retinopathy.

Since PPARγ2 is exclusively expressed in the adipose tissue, any effects of this variant on insulin resistance are secondary, pointing to the involvement of some intermediate link such as reduction in the release of free fatty acids from adipose tissue by suppressing lipolysis upon insulin stimulation. These reduced free fatty acid levels then result in more efficient glucose oxidation by muscle cells and decreased glucose production by liver cells in response to insulin [34-36], thus improving insulin sensitivity [9], which may subsequently ameliorate the pathophysiological basis of DR progression.

In addition, a link between the dietary habits and T2DM risk in relation to the Pro12Ala polymorphism was shown by Scacchi et al. [37], who found T2DM to be less prevalent among 12Ala carriers of populations who obtain more than 30% of their energy from dietary lipid intake, while those with less than 30% exhibited no clear pattern. The Pakistani population lies in the latter category where energy obtained from dietary lipids constitutes less than 30% of the total (FAO. Statistical Yearbook); therefore, in the Pakistani population, the possibility of such a link cannot be excluded in describing the relationship between dietary habits and the prevalence of T2DM and PDR among 12Ala carriers. In addition, in diabetics the protective effect of the 12Ala allele has been shown to be altered by the presence of another genetic variation, the T1431 synonymous SNP (rs3856806), in exon 6 of the gene [38]. The T1431 is in strong linkage disequilibrium with 12Ala, where the protective effects of the 12Ala allele are absent in individuals with the 1431T allele [39]. Therefore, such findings suggest a variable role of 12Ala in diabetes and its related complications, which requires further genetic elucidation in the Pakistani population.

In conclusion, we report a protective effect of 12Ala against PDR among individuals with T2DM. These effects were non-significant when compared to the subjects with NGNR, suggesting the putative relation of the phenotypic effects of this polymorphism with hyperglycemic conditions.
